# Silicon attenuates calcium deficiency by increasing ascorbic acid content, growth and quality of cabbage leaves

**DOI:** 10.1038/s41598-020-80934-6

**Published:** 2021-01-19

**Authors:** Dalila Lopes da Silva, Renato de Mello Prado, Luis Felipe Lata Tenesaca, José Lucas Farias da Silva, Ben-Hur Mattiuz

**Affiliations:** 1 Department of Agricultural Production Sciences, Soil and Fertilizer Sector, Faculdade de Ciências Agrárias e Veterinárias (FCAV), Universidade Estadual Paulista “Julio de Mesquita Filho” (UNESP), Via de Acesso Prof. Paulo Donato Castellane, s/n, Jaboticabal, SP 14884-900 Brazil; 2 Department of General and Applied Biology, Instituto de Biociências, Universidade Estadual Paulista “Julio de Mesquita Filho” (UNESP), Avenida 24 A, 1515, Rio Claro, SP 13506-900 Brazil

**Keywords:** Environmental sciences, Plant sciences, Plant development, Plant physiology

## Abstract

Calcium (Ca) deficiency in cabbage plants induces oxidative damage, hampering growth and decreasing quality, however, it is hypothesized that silicon (Si) added to the nutrient solution may alleviate crop losses. Therefore, this study aims at evaluating whether silicon supplied in the nutrient solution reduces, in fact, the calcium deficiency effects on cabbage plants. In a greenhouse, cabbage plants were grown using nutrient solutions with Ca sufficiency and Ca deficiency (5 mM) without and with added silicon (2.5 mM), arranged as a 2 × 2 factorial in randomized blocks, with five replications. At 91 days after transplanting, the plants were harvested for biological evaluations. In the treatment without added Si, Ca deficiency promoted oxidative stress, low antioxidant content, decreased dry matter, and lower quality leaf. On the other hand, added Si attenuated Ca deficiency in cabbage by decreasing cell extravasation while increasing both ascorbic acid content and fresh and dry matter, providing firmer leaves due to diminished leaf water loss after harvesting. We highlighted the agronomic importance of Si added to the nutrient solution, especially in crops at risk of Ca deficiency.

## Introduction

Calcium (Ca) deficiency hampers cabbage growth because this plant is a calcium-demanding brassica^1, 2^. Since Ca is absorbed only in the root meristematic regions, the small areas of the absorption surfaces require increasing the available Ca concentration in the culture medium to ensure adequate absorption^3, 4^. This characteristic of cabbage plants combined with the high demand for Ca may increase the risk of Ca deficiency in the crop.

Calcium, as calcium pectate, plays a structural role and is responsible for holding cell walls together in plants^3, 4^. Hence, Ca deficiency hinders the formation of cell walls, resulting in deformed leaves that affect the dry matter content^5^. At the molecular level, studies indicate that Ca deficiency in cabbage^6^ and broccoli^1^ causes oxidative stress because the plant loses cell defense signaling mechanisms responsible for eliminating the free radicals.

Thus, Ca deficiency increases the reactive oxygen species (ROS), inducing oxidative stress and causing damage to membranes while promoting the leakage of cellular electrolytes^7, 8^. Additionally, this oxidative damage degrades the cell wall^9^, impairs tissue firmness, and increases water loss which, in turn, results in leaf wilt^10^ and reduced shelf life of the harvested product.

Silicon (Si) may be an alternative to mitigate the biological damage caused by Ca deficiency in cabbage since it has been shown to increase the production of antioxidant compounds, such as ascorbic acid, in wheat^11^, chard and cabbage^12^. Studies in the literature have shown that Si decreases ROS content^12–14^ and, consequently, oxidative stress. Also, Si can act on the cell wall by combining carboxylic radicals while forming lignin and pectin cross-links^15^. The Si-enriched leaf tissues are firmer, thus decreasing the water flow from the leaves into the environment, keeping the tissues turgid for a longer period after harvest, improving leaf quality, as reported for lettuce without nutritional deficiency^14^. Thus, similar to Ca, it is possible to infer that Si could act on the cell wall of the cabbage leaf, providing firmer tissues.

Therefore, supplying Si to cabbage via nutrient solution can be an alternative to reduce the damage caused by Ca deficiency. However, the effects of providing Si to cabbage grown under Ca deficiency are still unknown. Additionally, studies focusing on supplemental Si are mostly carried out using foliar sprays^12, 16^ as the application method while the effects of Si supplied via nutritive solution are still little explored.

Hence, it is hypothesized that Si added to the nutrient solution attenuates the effects of Ca deficiency in cabbage by decreasing oxidative damage since the increasing ascorbic acid content favors the biomass accumulation and diminishes water loss after harvest, resulting in firmer leaves. If this hypothesis was to be accepted, adding Si to the nutrient solution should improve the production and quality of cabbage *cv*. Chato de Quintal, especially in those crops with some level of Ca deficiency.

## Materials and methods

### Experimental site and studied treatments

The experiment was conducted in a greenhouse of the Universidade Estadual Paulista, in Jaboticabal, Brazil, from March to June 2019. The experimental design consisted of randomized blocks, in a 2 × 2 factorial, as follows: Ca deficiency and sufficiency (5 mM), without and with added Si (2.5 mM), and five repetitions.

The Si source was sodium silicate and potassium stabilized with sorbitol (107 g L^−1^ Si; 16.44 g L^−1^ K_2_O and 60.7 g L^−1^ Na_2_O and pH 11.8). The potassium in the nutrient solution was balanced with KCl. In Ca deficient treatments, the nutrient solution Hoagland and Arnon^17^ was used at 10% of the ionic strength up 60 days after transplanting (DAT). After that, plants were deprived of Ca until harvesting.

### Plants and growing conditions

Cabbage *(Brassica oleracea* var. capitata) *cv.* Chato de Quintal seeds were sown in a 200-cell Styrofoam tray, filled with washed sand, and watered with distilled water twice a day until transplanting.

**Figure 1:**
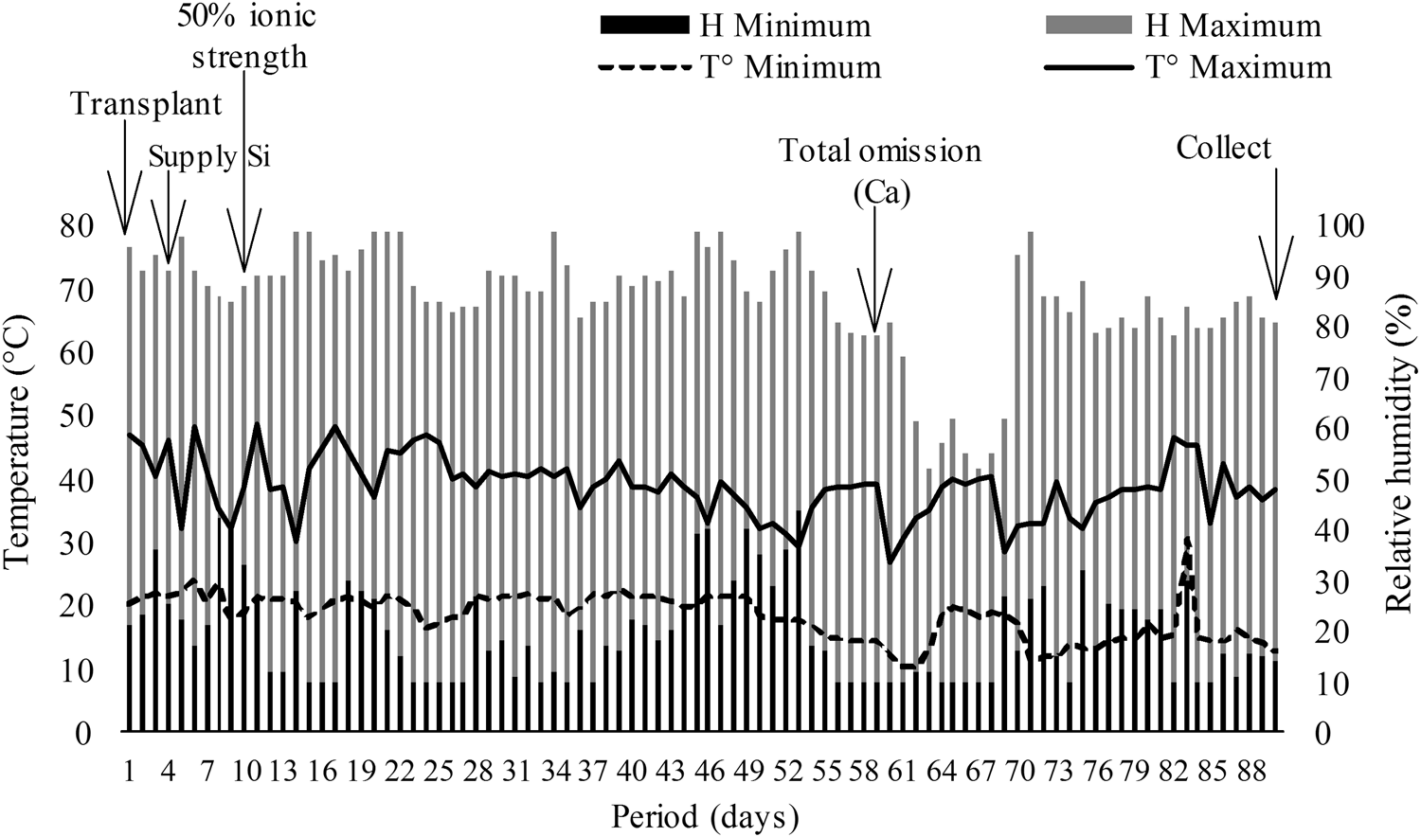
Maximum (T° Max) and minimum (T° Min) temperatures, and maximum (H Max) and minimum (H Min) relative humidity in the greenhouse, seedling transplanting date, Ca withdrawn date, and harvesting date of cabbage cv. Chato de Quintal.

At 26 days after sowing (DAS), the seedlings were transplanted into 1.7 dm^3^ polypropylene pots, filled with 1.5 dm^3^ of washed sand. Every pot with a cabbage plant consisted of an experimental unit. The sand was placed in a fine sieve, washed in running water, then saturated with a 1 mol L^−1^ HCl solution over 24 h to eliminate the organic matter residue. Subsequently, the sand was washed with deionized water to remove excess HCl. After transplanting the seedlings, greenhouse temperature and relative humidity were recorded daily using a thermo-hygrometer (Fig. 1). The average maximum temperature varied widely, 38.7 ± 21.2 °C, during the experimental period, above the optimal temperature. Cultivation at temperatures above 27 °C is known to cause stress to the plants^18^.

After transplanting, the complete nutrient solution and the respective treatments were applied via root, twice a day in the early morning and late afternoon. The solutions were prepared using distilled water deionized twice and checked for Si in the laboratory. The absence of Si deemed the solution suitable for the experiment.

The iron source of the modified Hoagland and Arnon^17^ nutritive solution was changed from Fe-EDTA to Fe–EDDHA, whereas Ca changed according to the studied treatments. The nutrient solution pH was adjusted daily to the target value of 5.5 ± 0.2, with HCL or NaOH accordingly.

In the first 10 days, the nutrient solution ionic strength was set at 25%, which was subsequently increased to 50%, remaining until the end of the experiment.

### Analysis

#### Electrolyte leakage index

At 91 DAT, six leaf discs (7.61 mm each) were removed from the third youngest leaf in the newly formed cabbage head to determine the rate of cell electrolyte leakage following the methodology in Dionisio-Sese and Tobita^19^. Subsequently, the leaf discs were immersed in 20 mL deionized water in a beaker at room temperature for 2 h. The solution electrical conductivity (EC1) was measure in a benchtop conductivity meter (TDS-3 digital meter). The samples were then autoclaved at 121 °C for 20 min, and after cooling, a new reading was performed to obtain the final electrical conductivity (EC2). The electrolyte leakage was determined as follows EC1/EC2 × 100.

#### Ascorbic acid

The ascorbic acid (AsA) content was quantified by titration with a 2.6 dichloro-phenol-indophenol sodium solution (Tillman’s reactive) and the results expressed as mg of ascorbic acid per 100 g FM (fresh matter)^20^. For extracting AsA, new and fully developed leaves from the cabbage middle region were mixed.

#### Leaf firmness index

Firmness was measured by inserting an 8 mm tip digital penetrometer to apply a force ranging from 5 to 200 N ± 1 N (Impac, Model IP-90DI, São Paulo, SP, Brazil) on the leaves of the cabbage third layer following the methodology proposed by Calbo et al.^21^. Three measurements were taken in the center of each leaf and expressed as Newton (N).

#### Accumulated fresh matter loss

The harvested cabbages were stored at 25 °C and weighed daily for five days on an electronic scale, ranging from 0.02 to 200 g ± 2 g (Marte® AL200C), to determine the accumulated fresh matter loss (AFML) in the evaluated period. The AFML was calculated based on the difference between the initial (first day) and final (fifth day) fresh matter weights, divided by the initial fresh matter (first day), and expressed as a percentage (%) as proposed by Chitarra and Chitarra^22^.

#### Fresh and dry matter of aerial parts and root dry matter

After harvesting, the aerial part was weighed to determine the fresh matter. Subsequently, cabbage and roots were washed in running water, then in a neutral detergent solution (0.1% v/v), HCl solution (0.3% v/v), and again in deionized water. After drying in a forced air circulation oven (65 ± 5 °C) to constant weight, the dry matter contents of aerial parts and roots were determined.

#### Ca and Si contents

The Ca content was determined using the dry samples previously ground in a Wiley mill following the methodology described by Bataglia et al.^23^ whereas Si content was determined from wet digestion according to Kraska and Breitenbeck^24^. The readings were performed in an atomic absorption spectrophotometer as described by Korndörfer et al.^25^. Finally, Ca and Si accumulated in the plant aerial part and roots were calculated based on the Ca and Si contents and dry matter.

### Statistical analysis

The data were submitted to analysis of variance by the F-test and, when significant, means were compared by the Student *t* test (LSD), at 5%, using SAS statistical software.

## Results

### Ca and Si accumulation on cabbage aerial part and roots

**Figure 2:**
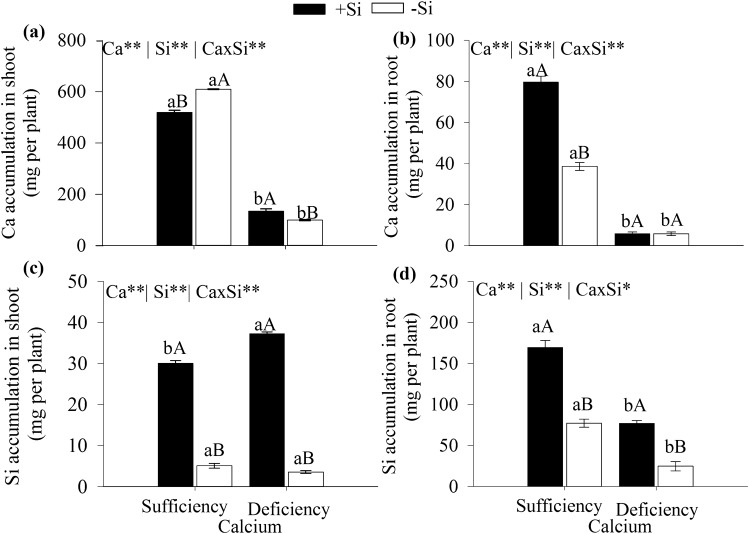
Ca accumulation in the aerial part (**a**) and root (**b**), Si accumulation in the aerial part (**c**) and the root (**d**) of cabbage grown under Ca sufficiency and deficiency, with (− Si) and without Si (+ Si). F test was applied: *(*p* ≤ 0.05) and **(*p* ≤ 0.01). The student *t* means comparison test was applied. Lowercase letters (**a**,**b**) indicate significant differences to calcium within the same level as Si (*p* < 0.05). Uppercase letters (**A**,**B**) indicate significant differences for silicon within the same level as Ca (*p* < 0.05). The bars represent the mean standard error. n = 5.

The highest calcium and silicon accumulations on cabbage leaves were observed in plants grown under Ca sufficiency treatments, with or without Si, indicating an interaction between these elements (Fig. 2a,c).

The added Si decreased Ca accumulation in the aerial part and increased in the root under Ca sufficiency (Fig. 2a,b) while increasing Ca accumulation in the aerial part under Ca deficiency (Fig. 2a). Further, the highest Si accumulation was observed in the aerial parts and roots of cabbage plants grown under Ca deficiency and sufficiency (Fig. 2c,d), respectively.

### Electrolyte leakage index and ascorbic acid content

**Figure 3:**
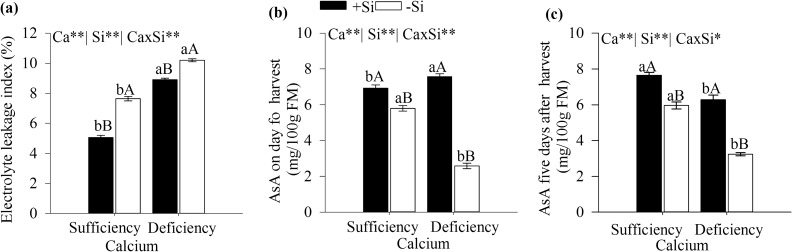
Electrolyte leakage index (**a**), ascorbic acid content on harvesting day (**b**), and ascorbic acid content (AsA) five days after harvesting (**c**) of cabbage grown under Ca sufficiency and deficiency, with (− Si) and without added Si (+ Si). F test was applied: *(*p* ≤ 0.05) and **(*p* ≤ 0.01). The Student *t* means comparison test was applied. Lowercase letters (**a**,**b**) indicate significant differences to calcium within the same level as Si (*p* < 0.05). Uppercase letters (**A**,**B**) indicate significant differences for silicon within the same level as Ca (*p* < 0.05). The bars represent the mean standard error. n = 5.

The electrolyte leakage index and the AsA content of cabbage leaves were affected by the Ca and Si interaction (Fig. 3). The electrolyte leakage rates were significantly higher in plants grown under Ca deficiency, with or without Si (Fig. 3a). However, the added Si decreased the extravasation rate of electrolytes from cabbage leaves grown under Ca sufficiency or deficiency.

The leaf AsA content is lower in cabbage grown under Ca deficiency, regardless of added Si. However, silicon supply increased vitamin C—AsA content in the leaf of cabbage under Ca deficiency on the harvesting day (Fig. 3b). Five days after harvesting, the treatment with added Si under Ca sufficiency resulted in cabbage leaves with the highest AsA content (Fig. 3c).

### Fresh matter of the aerial part and dry matter of the aerial and root parts

**Figure 4:**
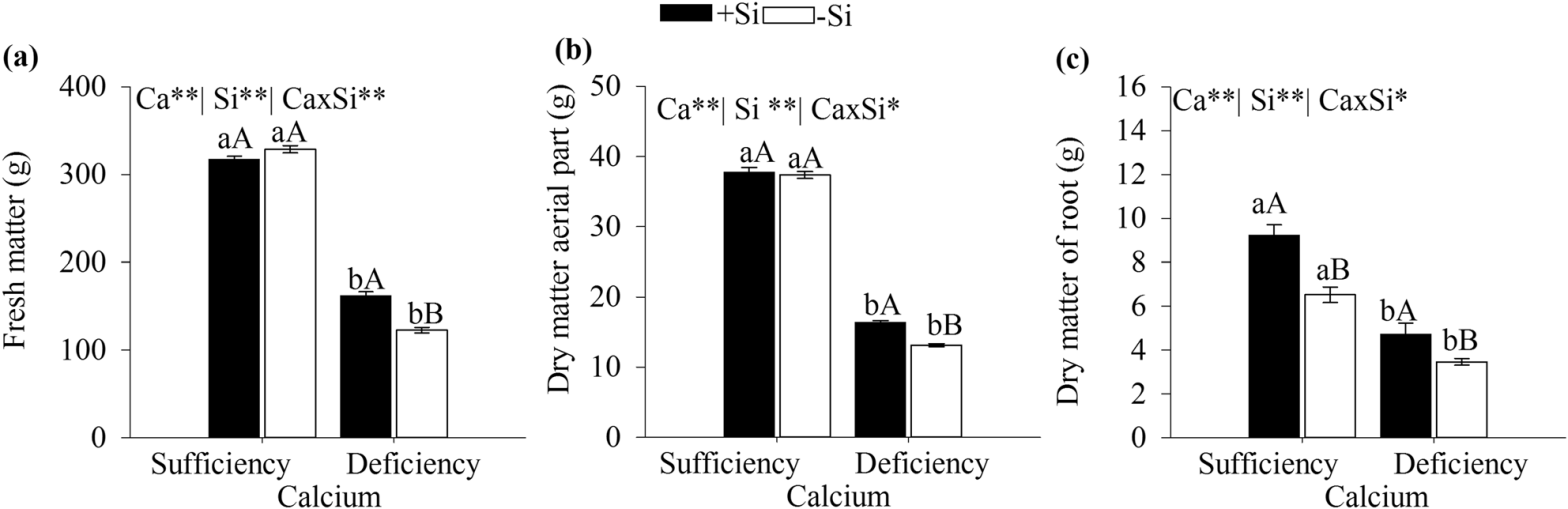
Fresh matter of the aerial parts (**a**), dry matter of the aerial parts (**b**) and roots (**c**) of cabbage under Ca sufficiency and Ca deficiency, with (− Si) and without added Si (+ Si). F test was applied: *(*p* ≤ 0.05) and **(*p* ≤ 0.01). The student *t* means comparison test was applied. Lowercase letters (a, b) indicate significant differences to calcium within the same level as Si (*p* < 0.05). Uppercase letters (**A**,**B**) indicate significant differences for silicon within the same level as Ca (*p* < 0.05). The bars represent the mean standard error. n = 5.

The fresh matter of cabbage aerial parts was affected by the calcium and silicon interaction, decreasing up to 37 and 31% in plants under Ca deficiency and Ca sufficiency, respectively (Fig. 4a). Under Ca sufficiency, the supplied silicon did not affect cabbage fresh matter.

**Figure 5:**
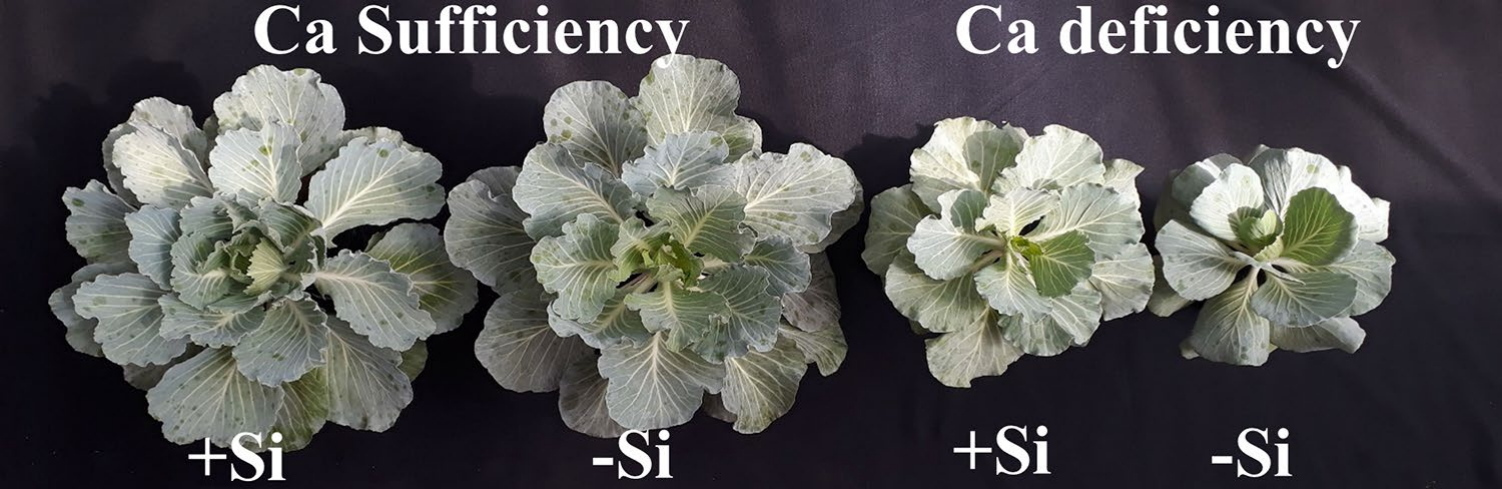
Cabbage plants grown under Ca sufficiency and Ca deficiency, without (− Si) and with (+ Si) added Si.

The dry matter of cabbage aerial part and root was significantly higher in plants under Ca sufficiency, with or without Si (Fig. 4b,c). But, the added Si promoted a greater dry matter accumulation in the aerial parts of cabbage under Ca deficiency (Fig. 4b). Indeed, the Ca and Si effects on the increasing dry matter of the aerial parts can be seen visually (Fig. 5) while highlighting that the added Si increased the root dry matter contents of cabbage plants grown under Ca sufficiency and deficiency.

### Accumulated fresh matter loss

**Figure 6:**
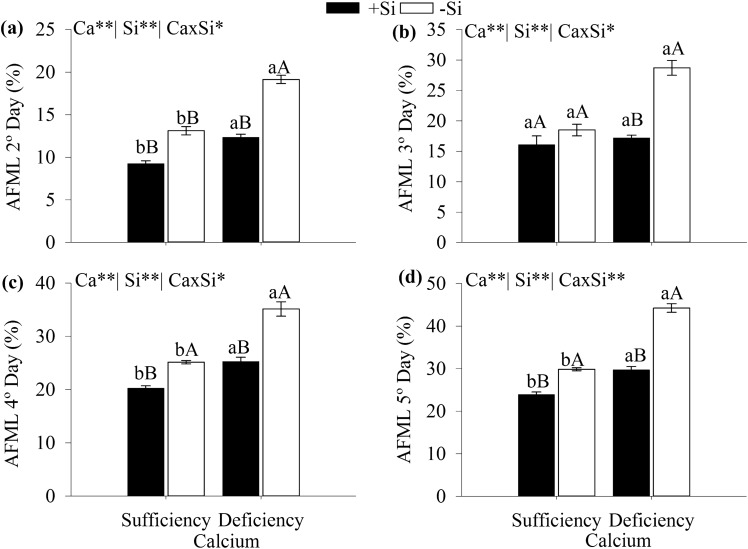
Accumulated fresh matter loss of cabbage under Ca sufficiency and deficiency, with (− Si) and without Si (+ Si), determined on the second (**a**), third (**b**), fourth (**c**), and fifth (**d**) day after harvesting. F test was applied: *(*p* ≤ 0.05) and **(*p* ≤ 0.01). The student *t* means comparison test was applied. Lowercase letters (**a**,**b**) indicate significant differences to calcium within the same level as Si (*p* < 0.05). Uppercase letters (**A**,**B**) indicate significant differences for silicon within the same level as Ca (*p* < 0.05). The bars represent the mean standard error. n = 5.

AFML was affected by the Ca and Si interaction five days after harvesting (Fig. 6). The AFML percentages were higher in cabbage under Ca deficiency without added Si on all evaluation days. Adding Si in the nutrient solution reduced AFML in cabbage plants grown under Ca-deficiency and sufficiency fifth days after harvesting.

### Firmness index

**Figure 7:**
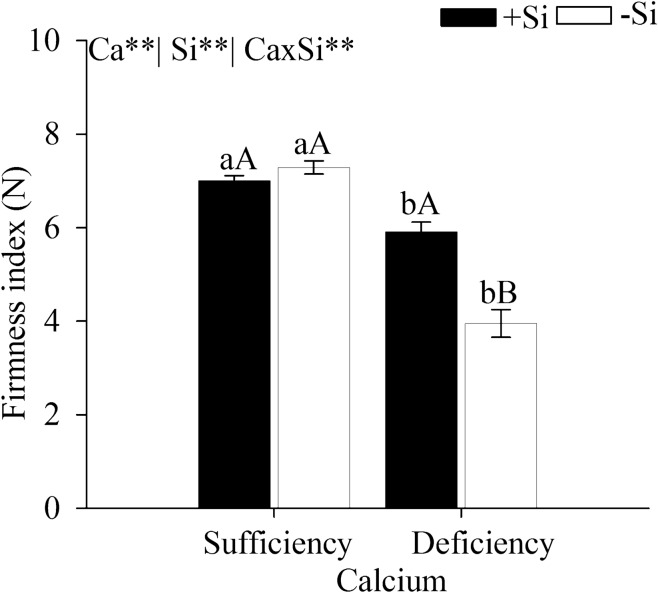
Leaf firmness index on the second day after harvesting of cabbage under Ca sufficiency and Ca deficiency, with (− Si) and without Si (+ Si). F test was applied: *(p ≤ 0.05) and **(p ≤ 0.01). The Student *t* means comparison test was applied. Lowercase letters (**a**,**b**) indicate significant differences to calcium within the same level as Si (p < 0.05). Uppercase letters (**A**,**B**) indicate significant differences for silicon within the same level as Ca (*p* < 0.05). The bars represent the mean standard error. n = 5.

The firmness index of cabbage leaves was affected by the calcium and silicon interaction. The leaf firmness index was significantly lower in cabbage under Ca deficiency, with or without Si (Fig. 7). However, the added Si increased the leaf firmness index in plants grown under Ca deficiency.

## Discussion

The Ca absorption rate decreased in cabbage grown under Ca deficiency but increased with added Si as demonstrated by the greater accumulation in the aerial part (Fig. 2a). This result indicates a synergistic effect of Si on the increase of Ca absorption, possibly because Si favors the growth rate of the root system^26^ and the molecular effect of Si on the increasing ATPase activity in the membranes involved in Ca absorption^27, 28^. To our knowledge, this report on the benefit of Si in cabbage grown under Ca deficiency is unprecedented since our literature review showed no reports on this subject.

However, added Si also increased Ca absorption by the aerial parts of other plants grown under Ca sufficiency, as previously reported for corn by Kaya et al*.*^29^; wheat, Mali and Aery^30^; broccoli and cauliflower, Barreto et al.^13^; and yellow passion fruit seedlings, Silva Júnior et al.^31^. These results show the importance of further studies aiming to understand the molecular mechanisms of the beneficial role Si plays in the absorption of other cations such as Ca.

The decreasing Ca absorption in Ca deficient plants compared to Ca sufficient, without Si, resulted in increased electrolyte leakage rate and decreased AsA content (Fig. 3). This result demonstrates that Ca deficiency increases the activity of pectolytic enzymes (polygalacturonase)^32^ and lipid peroxidation due to increased malondialdehyde^10^, which aggravated by decreasing antioxidants (ascorbic acid), promoting degradation of membrane compounds and cell wall damage, increasing the electrolyte leakage in turn^33^.

The increasing stress observed in Ca deficient plant tissue, without Si, decreased plant growth and hampered the production of fresh and dry matter of aerial parts and roots (Fig. 4). Similarly, this behavior has been already reported for brassica cabbage^33^, cauliflower^34^, and other leafy vegetables such as lettuce^35^ and basil^36^.

Besides, it was evident that Ca deficiency increased the AFML between the second and fifth days after harvesting (Fig. 6), and this increased water loss from the plant tissue can decrease vegetable shelf life. Likewise, this high water loss after harvesting through the leaves of plants grown under Ca deficiency has been previously reported for other leafy vegetables, more specifically in spinach leaves by Chao et al.^10^. The high AFML becomes evident as leaf wilts, and turgidity and firmness of plant tissue diminish as observed in the plants grown under Ca deficiency in this study. Ca plays an important structural role in plants by conferring rigidity to the plant tissue since it constitutes the middle lamella and cell wall Taiz et al.^37^ while the increasing transpiration on the leaf surface of plants grown under Ca deficiency^10, 32, 38^ decreases the water content Chao et al.^10^, turgidity and firmness of plant tissue.

Additionally, the results show that cabbage plants absorb Si, given the Si increase observed in the aerial parts and roots of plants grown under Ca sufficiency and deficiency (Fig. 2c,d). The Si content reached 1.6 g kg^−1^ (data not shown) in the aerial parts of plants under Ca sufficiency which (Fig. 2c), according to^39, 40^, indicates a non-accumulating plant since the Si content is less than 5 g kg^−1^. Non-accumulating plants are those that accumulate more Si in the roots than in the aerial parts, possibly due to the low activity of the Lsi1 and Lsi2 transporters in the membranes of the root cells^41^. Similar behavior was observed in this study since Si added to cabbage grown under Ca sufficiency increased Si accumulation in the roots (170 mg per plant) (Fig. 2d) compared to the aerial parts (30 mg per plant) (Fig. 2c).

Clearly, Si added to cabbage under Ca deficiency exhibited decreased extravasation rate of cellular electrolytes and plant tissue with increased AsA content (Fig. 3). This Si benefit may be attributed to several factors such as the indirect effect of increasing Ca absorption (Fig. 2a) and the direct effects of increasing antioxidant compounds and ascorbic acid (Fig. 3b,c). There are reports in the literature on the decreasing leakage of cellular electrolytes ensuring the maintenance of tissue integrity for several species such as wheat Ma et al.^11^, chard and cabbage, via Si foliar spraying^12^, broccoli and cauliflower^13^, and corn^33^ but no reports on cabbage.

The added Si lessened the effects of Ca deficiency by reducing plant stress as indicated by the decreased leakage of electrolytes (Fig. 3a), promoting cabbage growth given the increased fresh and dry matter of the aerial parts and roots (Fig. 4). This unprecedented finding shows that added Si reduces the nutritional stress of cabbage grown under Ca deficiency, becoming especially important in brassica since Ca deficiency is common in the culture, limiting its growth^5^.

Furthermore, added Si improved the quality of leaves of cabbage grown under Ca deficiency after harvesting since the AFML decreased between the second and fifth days post-harvesting (Fig. 6). On the second day after harvesting, the leaves of cabbage under Ca deficiency, with or without Si, were unsuitable for commercialization since AFML was greater than 10% (Fig. 6b). Further, Si added in the nutrition solution of plants under Ca sufficiency, resulted in 9% AFML, within the acceptable limits for commercialization, according to Chitarra and Chitarra^14^. This result shows the importance of Ca and Si for improving the quality of vegetables such as cabbage produced for human consumption.

On the fourth and fifth days post-harvesting, the AFML results indicate that Si added in plants under Ca sufficiency improved cabbage quality in the period (Fig. 6c,d). The post-harvest Si effect has already been reported for lettuce^14^, and chard and cabbage^12^ grown without nutritional stress.

This reduced AFML (Fig. 6) and greater firmness of the plant tissue (Fig. 7) with added Si is possibly associated with the pectin polymers as seen in rice plants^42–44^. Si acts on the hemicellulose and lignin synthesis, by increasing the components of the cell wall in rice plants^15, 42^, reducing the activity of enzymes that degrade cell wall^33^ and decreasing tissue degradation due to increased AsA content and antioxidant action^45^.

Also, the increased ascorbic acid content (Fig. 3b,c) is highly desirable due to its antioxidant action and for being a vitamin (C) source, essential for human health^46^, while indirectly promoting biofortification and better nutritional quality beyond the firmness.

Our research shows that added Si is beneficial to cabbage plants grown under nutritional stress, corroborating the literature reports that Si is more beneficial to plants grown under stress compared to stress-free crops^13, 31, 47^.

## Conclusions

The Si added in the nutrient solution supplied to cabbage plants increased the contents of vitamin C and biomass while reducing leaf water loss and, thus, ensuring longer storage time post-harvest. This finding highlights the agronomic importance of adding Si to the nutrient solution used in the cabbage crop, especially those crops at risk of Ca deficiency.
